# Barriers and Facilitators to Medication Adherence among the Vulnerable Elderly: A Focus Group Study

**DOI:** 10.3390/healthcare12171723

**Published:** 2024-08-29

**Authors:** Martina Horvat, Ivan Eržen, Dominika Vrbnjak

**Affiliations:** 1 National Institute of Public Health, 1000 Ljubljana, Slovenia; ivan.erzen@nijz.si; 2 Faculty of Health Sciences, University of Maribor, 2000 Maribor, Slovenia

**Keywords:** medication adherence, elderly, vulnerability, barriers, facilitators

## Abstract

Poor medication adherence is a significant public health issue, especially among the vulnerable elderly, leading to increased morbidity, mortality, and healthcare costs. This study aimed to explore, identify, and understand the barriers and facilitators to medication adherence among vulnerable elderly individuals. We conducted a qualitative study using focus group interviews with 31 participants, including community nurses, social care services, volunteers from non-governmental organizations, patient association members, and informal caregivers, using semi-structured questions and inductive content analysis to gather and analyze qualitative data. Two main categories, “Perceived barriers” and “Facilitative interventions” were developed. The findings revealed multiple barriers, including medication-related barriers, patient-related barriers and barriers related to the healthcare system and healthcare personnel. Participants also highlighted the importance of facilitating interventions like medication management, health education, supportive social networks, and ensuring continuity of care. The study underscores the need for targeted strategies to improve medication adherence among the vulnerable elderly.

## 1. Introduction

Poor medication adherence is recognized as a global public health issue significantly impacting the management of both chronic infectious and non-infectious diseases [1,2]. Adherence to medications is widely studied in the literature, as it is a detrimental factor in reducing the mortality and morbidity in chronic diseases and, also, in reducing hospitalization total healthcare costs [3]. Medication non-adherence is associated with almost 200,000 deaths annually and €80–125 billion in the European Union [4]. Medication use is still a serious problem among older adults in the OECD countries [5]. Poor adherence to antibiotic regimens also exacerbates infections and contributes to the development of antimicrobial resistance (AMR) [6].

The share of the aging population is increasing, and with it, the number of elderly individuals needing medication at home [7]. Non-adherence is particularly problematic among the elderly due to multiple chronic conditions, a greater number of different drugs, and, consequently, greater health-related risks [2,8]. The vulnerable elderly are especially at risk, as their ability to self-medicate is often diminished by limited autonomy and other factors such as reduced cognitive abilities, education, resources, and physical strength [9]. While vulnerability is not specific to any age group, the elderly are more likely to face greater risks and have a reduced capacity to respond [10]. Vulnerability occurs when adaptive capacity falls below the level needed to maintain quality of life or prevent premature death [10]. Definitions of vulnerability in the elderly vary, but generally include conditions like multimorbidity, functional disability, and significant socio-economic or psychological challenges that increase the risk of hospitalization or institutional care [11]. The vulnerable elderly may live in a variety of settings, including their own homes, assisted living facilities, nursing homes, or hospice care. It is also important to recognize that elderly individuals may perceive their vulnerability differently than healthcare professionals [12].

The elderly are often prescribed several medications due to their health conditions, which makes it difficult for them to be self-sufficient in taking their medicines at home [7,13]. Numerous factors influence non-adherence, ranging from patient and healthcare-provider-related issues to systemic issues [14,15,16,17]. Dharvees et al. [18] identified barriers to medication adherence such as forgetfulness, lack of awareness, poor literacy, and complex regimes. Facilitators included good access to the healthcare system, patient counseling, regular monitoring, and medication refills. Dijkstra et al. [19] observed issues with improper medication storage and management, underscoring the need for better healthcare support in medication self-management. Lehmann et al. [20] categorized barriers into the following five major groups: disease-related; medication-related; healthcare system-related; demographic and socioeconomic factors; and patient and caregiver factors. Forsyth et al. [21] highlighted several barriers, including comorbidity and socio-economic factors, recommending interventions that enhance patient trust and simplify treatment regimes.

To ensure the effectiveness of these interventions, it is essential to have a comprehensive understanding of both barriers and facilitators, especially with respect to the vulnerable elderly [1]. Interventions must focus on identifying specific patient barriers, addressing broader adherence challenges, designing strategies to mitigate these barriers, and incorporating tailored strategies and sustained social support [22].

Therefore, this study’s aim was to explore, identify, and understand the barriers and facilitators to medication adherence among the vulnerable elderly.

## 2. Materials and Methods

### 2.1. Study Design

A qualitative study was conducted using focus group interviews, employing the Consolidated Criteria for Reporting Qualitative Research for reporting [23]. This study is part of a larger mixed-methods study aimed at developing and evaluating the effectiveness of a set of interventions by community nurses for the vulnerable elderly to improve medication adherence.

### 2.2. Setting and Participants

The first author recruited participants for the focus groups through purposive sampling by personal invitation by the first author. The inclusion criteria included professionals, volunteers, and patients who provide care or support to vulnerable elderly individuals or are themselves vulnerable elderly living at home. In Slovenia, 95% of all elderly people live in their own homes, meaning that the majority of vulnerable elderly individuals live in their home environments. They have access to home care services; within primary healthcare, their care involves community nursing providers, family doctors working alongside teams in family medicine clinics, and specialists from mental health centres who may also participate in their care [24,25,26]. Community nurses autonomously and independently plan and offer health promotion and preventive care services at home and in the community. In collaboration with specialists in family medicine, paediatrics, and gynaecology, they also implement diagnostic and therapeutic procedures at the patient’s home [26,27,28,29]. Therefore, we have invited community nurses and home care providers from social care services, who work with vulnerable elderly individuals, volunteers from non-governmental organizations focused on supporting vulnerable elderly groups, such as the Union of Pensioners’ Societies, the Red Cross, Caritas, members of patient associations with chronic diseases, and informal caregivers (relatives or significant others of the patients).

A total of 31 individuals were invited and participated in four focus groups, each ranging from 6 to 11 participants. None of the participants refused to participate or withdrew from the study.

Focus groups were held at predetermined times in various locations, including the Nurses, Midwives, and Health Technicians Association premises, the Union of Pensioners’ Societies, and the Elderly Home Care. The composition of the focus groups was homogeneous, taking into account the position and activities of the participants. Focus group 1 included eight home care providers, including seven women and one man, with an average age of 43 years. Focus group 2 included six community nurses, all women, with an average age of 52.2 years. Focus group 3 included six volunteers from non-governmental organizations focused on supporting vulnerable elderly groups, all women, with an average age of 70.7 years. Focus group 4 included 11 members of patient associations, patients with chronic diseases, seven women and four men, with an average age of 73.5 years.

### 2.3. Data Collection and Analysis

Focus groups were moderated by the first author, a female PhD candidate and a community nurse by profession, under the supervision of two experienced researchers in public health and nursing (PhD, professor and associate professor, male and female). An assistant was also present to provide technical support (female). The moderator kept notes and observations during the focus group. The sessions were organised between June and July 2021. They were conducted as semi-structured interviews. The interview guide was developed iteratively by authors based on a literature review focused on barriers and facilitators to effective medication use among the vulnerable elderly, knowledge about prescribed medications, and strategies to enhance medication adherence [10,14,17,30,31,32,33,34,35,36,37,38,39] and clinical expertise (see Supplementary Materials File S1). Each focus group started with an explanation of the study’s objectives, the definition of the elderly vulnerability of the and the completion of consent forms by all participants, who were also provided with a printed copy of the interview questions.

Each focus group lasted approximately 75 min. The interviews were recorded and transcribed verbatim, resulting in a transcript of 162,325 characters, without spaces. The inductive content analysis method by Elo & Kyngäs [40] and Kyngas [41] was utilized for the qualitative data analysis. The process involved open coding, category formation, and abstraction to formulate a comprehensive description of the investigated topic through category development. Each quote was marked with the number of the focus group and the participant, with the first number representing the focus group and the second representing the participant.

### 2.4. Trustworthiness

The study adhered to Lincoln and Guba’s criteria [42] for trustworthiness, encompassing credibility, dependability, confirmability, and transferability. Credibility was established by exact transcription of the interviews and independent data analysis by two researchers. Dependability was supported by detailed documentation of the sampling, data collection, and analysis processes. Confirmability was ensured through ongoing review and refinement of data to verify reproducibility by other researchers. Direct quotes from the focus group participants underpinned the interpretation of findings. For transferability, the authors provided comprehensive descriptions of the study’s context, profiles of focus group participants, sampling methods, and data collection sites, aiming to affirm the relevance of the findings to other settings. Furthermore, the interview guide was reviewed and revised by ethics experts and the research team prior to data collection, strengthening the study’s overall integrity.

### 2.5. Ethical Considerations

Prior to conducting the study, we obtained approval from the National Medical Ethics Committee of the Republic of Slovenia, Number: 0120-114/2021/3. Participants voluntarily and anonymously joined the study, and they were informed about the study’s procedures before signing the informed consent. They were also informed of their right to stop the recording or withdraw from the study at any point.

## 3. Results

Two main categories, “Perceived barriers” and “Facilitative interventions” were developed (Table 1).

### 3.1. Main Category Perceived Barriers

This category included medication-related barriers, patient-related barriers and barriers related to the healthcare system/healthcare personnel.

#### 3.1.1. Medication-Related Barriers

Participants reported several barriers related to medication instructions, difficulties in procuring, switching, and replenishing medication supplies, purchasing non-prescription drugs and dietary supplements, polypharmacy/excessive medications, and problems with improper medication handling and refusal/discontinuation of medication. Medication instructions are often too complex and written in small print, making them hard for the vulnerable elderly to read and understand. The vulnerable elderly often forget to renew their medication supplies, leading to interruptions in treatment. Health insurance rules and the mismatches between prescription periods and packaging quantities cause further confusion. The vulnerable elderly are also vulnerable to aggressive advertising and often buy unnecessary supplements, leading to financial strain and potential health risks without consulting healthcare professionals. Taking multiple medications increases the risk of improper use, leading to health deterioration. Changes in medication regimens for the vulnerable elderly when they develop a new illness also presents a barrier. The vulnerable elderly often struggle with managing the timing and dosage of various drugs. They often need help preparing medications and may take them irregularly or hide them. Self-adjustment of therapy and non-compliance with prescriptions are common issues. Participants also noted that some vulnerable elderly patients take multiple medications for the same indication (e.g., sleeping pills, tranquillizers, painkillers), which increases the risk of falls.

*“…The problem is that those who can still remember and want to read the instructions can’t because the print is so small…” (3/3)*

*“Some people here also stock up, right… for example, when they are given those nice pills, they quietly put them in their pocket.” (1/8)*

*“… Sometimes people have a lot of medication at home, whole bags full” (2/4)*

*“…A multitude, really a multitude of medicines they have, and then they also buy what is advertised on TV…” (2/5)*

*“I have to say that what particularly bothers me is this propaganda, as the previous speaker mentioned, those alternative pills they advertise on television, and… people just… we buy this, we buy that… and… /…/ we fall for it, yes, don’t we.” (4/11)*

*“…The problem if you take more medicines, like four pills in the morning, and you’re always in doubt which to take first, what the time gap should be…” (4/8)*

*“…I have one person who injects insulin whenever they feel like it, regardless of the instructions…” (2/1)*

#### 3.1.2. Patient-Related Barriers

Participants reported the following patient-related barriers: physical difficulties and conditions; decline in general fitness and cognitive abilities; and health literacy and ICT literacy. Health conditions significantly affect medication adherence, especially with multiple concurrent illnesses. Common barriers include issues with vision, hearing, understanding, swallowing difficulties, and tablet size. Loss of thirst sensation, challenges with applying drops or suppositories, and tremors or joint pain complicate opening medication bottles and blister packs. Routine disruptions, such as hospitalizations or injuries, further complicate medication adherence. Medication adherence is influenced by the overall health and fitness of the elderly. Cognitive decline increases vulnerability and barriers, with patients often forgetting to take medications, taking them incorrectly, or not recognizing the benefits of the medications. The vulnerable elderly often lack knowledge about their medications, including indications, administration methods, and side effects. They frequently identify medications by color and shape rather than name. Family members assisting with medication also lack understanding, leading to improper administration. Instructions are often not clearly explained by doctors or pharmacists. Additionally, the vulnerable elderly struggle with understanding health information and using e-health systems like e-prescriptions. In some cases, patients or their relatives adjust the therapy on their own (“self-medicating”) or discontinue medications without consulting a doctor (“because they don’t help”). Participants also observed that despite using pill organizers, the vulnerable elderly take their medications irregularly, either occasionally or all at once for the day. In other cases, they hide the prepared medications, in, for example, a pocket, and do not take them.

*“…sometimes antibiotics are prescribed, but the person is too weak to swallow large tablets. Doctors should consider the patient’s condition and prescribe accordingly…” (2/5)*

*“… Forgetfulness is one of those things, especially when there are many pills, right… for example, we might tell ourselves, I will, I will, I know this, and so on, right… and actually, it comes to the point where, for instance, you forget to take some pills…” (4/10)*

*“…they can get confused with dosette boxes, taking all the day’s pills at once…” (2/4)*

*“They get medications but aren’t properly informed on how to take them. For instance, some medications shouldn’t be crushed…” (1/6)*

*“Many don’t know what their medications are for…” (1/3)*

*“From my late grandmother, I can say that she also had difficulty opening the medication… in the end, she opened all of them, mixed all the medications together… in the end, we no longer knew what what was…” (3/5)*

*“… they found out that I need to take medication for blood pressure… and I took it for a while, then for 3 years, I… I stopped taking that medication… for three years or two. And not even half a year passed, and I had a stroke… the first, the second, the third… because of the medication I stopped taking…” (4/1)*

#### 3.1.3. Barriers Related to the Healthcare System/Healthcare Personnel

Poor communication between healthcare providers at different levels leads to issues with continuity of care and information transfer. The vulnerable elderly, or their relatives, often do not visit their personal physician after discharge to review medications and any changes made in the hospital, leading to improper medication use, worsening health, and potential rehospitalization. Discharge often occurs when personal physicians are unavailable (e.g., Friday afternoons, weekends), leaving patients to be visited by home care nurses who struggle to obtain accurate patient information. Sometimes, patients are discharged without relatives or home care services being notified, leaving them unsupported. Discharge summaries given to patients or relatives often contain incomplete or unclear instructions. Patients and their relatives are rarely educated about new medications or therapy changes at discharge. Participants highlighted the inaccessibility of personal physicians, which worsened during the COVID-19 pandemic and continued afterwards. The vulnerable elderly struggle with phone communication (busy lines, unanswered calls, confusing automated responses), making it difficult to schedule doctor appointments or establish direct contact. Phone communication for medication orders and prescription renewals is problematic and poses a significant barrier. Participants noted that family medicine doctors often have too little time to talk with patients. Frequent changes in healthcare providers or a lack of consistent follow-up can lead to gaps in care, particularly for the vulnerable elderly with chronic conditions. This disruption often results in inadequate monitoring and management of their health needs. This inconsistency in pharmacist engagement further complicates the continuity of care.

*“…some have these things really well organized, while others come from the hospital with discharge papers and such but don’t go to the doctors to review the therapy, whether it’s new or modified, right…” (1/1)*

*“…when new medications are prescribed in the hospital, someone should inform the relatives or the patient about what to discontinue and what changes have been made…” (2/1)*

*“Some don’t manage at all, even if they have a phone at home. They get confused by the automated systems and just hang up. They can’t reach their doctor, so they ask us to call for them…” (2/6)*

*“… let me tell you, when it comes to medication, the pharmacist is crucial… they have studied this, just like doctors… and pharmacists should… some give more advice, some don’t… something should change here… I have felt the need a hundred times in the pharmacy, for example, to consult with someone, but there’s no one… they just give you the medicine like in a store… and then you’re on your own…” (4/8)*

### 3.2. Main Category: Facilitative Interventions

This category included medication management, health education, supportive social networks and ensuring continuity of care.

#### 3.2.1. Medication Management

Participants questioned who should be responsible for managing medications. They believe that personal physicians should review and organize the medication regimens for the vulnerable elderly. A community nurse can prepare a list of medications that the patient has at home, while the physician ensures they match the patient’s medical records. The physician should also check the compatibility of newly prescribed and existing medications and update the medication list after hospital discharge. An organized medication list with simple indications for each medication would help the vulnerable elderly to follow their doctor’s instructions. Different colors or symbols for different times of the day could be used for clarity. In the case of hospitalization, having a printed medication list would be beneficial. Participants suggested that pharmacies could provide a simplified medication summary with large text, colors, and key information to prevent incorrect medication use. Participants recommend that all patients obtain a suitable medication dispenser based on the number of medications they take, with different parts of the day marked in various colors. Only one person should be responsible for preparing the medications. Other medications should be removed and stored elsewhere to avoid confusion. Educating the patient and their informal caregivers about preparing medications according to the list and encouraging them to follow it is important.

*“… my partner, who goes to a specialist, and sometimes the medications prescribed by the specialist and the treating doctor conflict, this should not happen…” (4/11)*

*“…having a list, some had it when they went to the hospital or for an appointment, their personal doctor gave them a complete list… if they had it at home, maybe color-coded, morning is this color, noon that color, evening that color…” (2/3)*

*“…I have a suggestion… it would help if each patient had a sheet with the purpose of the medication, its benefits, and possible harms, written in large letters and highlighted with one color… some patients want to know about their medications but can’t read the small print, so a summary with the core information highlighted would be useful…” (3/3)*

*“…I had an idea that in the family, someone should be designated to pack the medications… everything else should be removed because I’ve seen… they have prepared medications, but if there are boxes around, some patients will still take from those too… so sometimes they take from both…” (2/6)*

*“… community nurse would then give me medication… in my opinion… well, this is my thought… this is the solution because we can’t always do what is common nowadays: ‘if there’s no one to give the medication, they should go to a nursing home…” (4/2)*

#### 3.2.2. Health Education

Participants emphasized the importance of educating the vulnerable elderly and informal caregivers about proper medication use and management. Often, there is a lack of understanding about correct medication administration; patients struggle with changes. Physicians should educate patients on prescribed medications, while community nurses should ensure that instructions from doctors and pharmacists are understood, including dosage and timing. They should verify that necessary health measurements are conducted correctly and remind patients to renew supplies on time and to store medications safely. The vulnerable elderly should seek information from professionals, not the Internet or social media. An emphasis should be on medication efficacy over side effects, and regular intake. Written materials should contain clear and simple information written in capital letters.

*“They don’t understand… when we visit, we explain it in simple terms, unlike the medical jargon used in hospitals… so they can grasp it better.” (2/3)*

*“It’s important they understand, for example, ‘If you don’t take this, your condition will worsen. If you do take it, watch out for this side effect’. They need to weigh the benefits against the drawbacks.” (3/3)*

*“There is also a problem, for example, with the elderly that I notice, particularly those who have retention issues… some of them don’t want to take the pill because then they will urinate a lot, right… and then they get wet, and now it’s hot… so they tend to skip taking some pills, right… but then this leads to other health complications, right… and then a lot of explaining is needed.” (1/6)*

*“… It’s written in such a long way, and then the side effects, and usually, you don’t go through everything…you need support from professionals.” (4/7)*

*“Instructions could be clearer, not just for elderly, but for everyone. Medical terms are hard to understand, so people end up reading about side effects on forums online, which are often misleading.” (3/5)*

#### 3.2.3. Supportive Social Networks

A supportive social network is vital for the vulnerable elderly, aiding medication adherence and allowing them to stay in their homes longer. Family and neighbors provide significant support, and maintaining good relationships is crucial. Participants believe that nearby relatives should assist the vulnerable elderly with their medications. When the vulnerable elderly cannot manage on their own, family, or other supporting individuals should provide oversight and assistance. Rural communities often organize local help, while urban areas see more isolation of vulnerable elderly people. NGOs and volunteers offer companionship and practical assistance, sharing valuable information and experiences with the elderly. Volunteers can help them with medication inquiries and offer lay support. Patient associations also play a significant role, with members sharing experiences, information, and mutual support.

*“Many vulnerable elderly are left to fend for themselves… often because younger people are not interested in helping…” (2/1)*

*“In rural areas, community support is more common. For example, a woman from the local community took responsibility for a vulnerable elderly man’s medication management, and later, they even arranged for a caregiver and meal delivery…” (2/6)*

*“I think family members who are nearby should take responsibility for their elderly relatives’ medication. It costs nothing to visit your mother and ensure she is taking her medication correctly.” (1/1)*

*“… without our association, I can’t imagine how it would be, right… We also talk about medications, what kind of medications someone is taking… and those things, right…” (4/1)*

*“…otherwise, we also visit the elderly during certain occasions; New Year’s and so on… mainly, they need a word, a conversation, as they say… a kind word, a smile… offering that to them, talking with them, it means a lot to them, right… otherwise, even if not in person, we try to keep in touch over the phone or something like that…” (3/1)*

#### 3.2.4. Ensuring Continuity of Care

This category included ensuring proper information transfer among providers upon patient discharge, helping patients to establish contact with their personal physicians and coordination and cooperation among all involved in care. Timely transfer of all relevant information is essential to ensure continuity of care. Participants emphasized the need for a standardized medication regimen management system. They suggest that information transfer should be systematized at the national level through modern communication methods (e-health). Participants proposed empowering the vulnerable elderly and their informal caregivers to use modern technologies for remote communication. They highlighted that community nurses act as coordinators for all forms of home assistance and as the link between the vulnerable elderly and their personal physician. Coordination among all caregivers is crucial for the effective care of the vulnerable elderly. Participants noted that the function is interdependent, requiring proper distribution of help and support throughout the day and week.

*“For example, when a nurse sees a patient in general practice or in the hospital and notices something uncertain, they could alert us to make a home visit at the same time, to check on the patient.” (2/1)*

*“…then you phone the surgery, the GP, and the nurse says; now you’re going to send us more of these?…, that is, they’re overbooked, but …” (2/5)*

*“…sometimes you phone the doctor three days before, and he doesn’t finish the prescription, so the drugs are running out, and sometimes it gets disconnected, doesn’t it…” (2/4)*

*“Community nurses and home assistance can come every day if needed… if there’s no other option.” (4/2)*

## 4. Discussion

The aim of this study was to explore, identify, and understand the barriers and facilitators to medication adherence among the vulnerable elderly. The findings highlighted a range of challenges faced by the vulnerable elderly, including medication-related barriers, patient-related barriers, and systemic healthcare barriers, as well as potential interventions to improve adherence, which included medication management, health education, supportive social networks and ensuring continuity of care.

Participants highlighted issues such as complex medication instructions, difficulties in procuring and switching medications, polypharmacy, and improper medication handling. These barriers are exacerbated by factors like the small print on medication labels, aggressive advertising, and inconsistent health insurance policies. Prior research supports these findings. Amorim et al. [43] emphasized that the elderly often misunderstand medication instructions due to polypharmacy, poor literacy, and memory issues. Similarly, Dharvees et al. [18] noted barriers such as forgetfulness and lack of awareness, suggesting improved patient counseling and regular monitoring as solutions. Dijkstra et al. [19] highlighted improper medication storage and handling, underscoring the need for healthcare workers to assist in medication management. Participants pointed out that one of the problems is the purchase of non-prescription medicines and food supplements. The findings suggest that people living with chronic diseases and taking multiple medicines, including non-prescription medicines, are likely to be non-adherent to prescription medicines [44].

Physical difficulties, cognitive decline, and low health literacy were major barriers. Issues, such as vision and hearing impairments, difficulty swallowing, and tremors, complicate medication adherence. It is very important to identify the barriers related to an elderly person’s physical abilities. A higher level of frailty among elderly patients may be considered as a determinant of lower adherence [45]. Poorer vision and manual dexterity are associated with poorer self-management of medication [46]. Problems associated with opening the packaging of medicines represent a barrier to medication adherence that is often neglected, even though it can lead to unintended treatment discontinuation [47,48]. Cognitive decline leads to forgetfulness and improper medication use. Additionally, Hyvert et al. [49] emphasized the influence of beliefs about medications and self-efficacy. Holt et al. [50] also underscored the importance of memory, knowledge, and social support in medication adherence. Memory function has a significant impact on medication adherence; therefore, intervention planning should focus on activities that increase memory ability [51]. Coskun et al. [52] found that improving health literacy significantly increases medication adherence, highlighting the need for effective educational programs.

Poor communication between healthcare providers, especially during patient discharge, and the inaccessibility of personal physicians were significant barriers. These issues lead to improper medication use and potential rehospitalization. Medication reconciliation during the hospital discharge process is of paramount importance and must be supported by coordinated, effective communication between all healthcare professionals involved in the hospital discharge process, taking into account patients, family members and carers, and encouraging their active participation [53]. Slovenia has only recently begun integrating clinical pharmacists at the primary level of the healthcare system (e.g., health centers and family medicine clinics) in contrast to the secondary and tertiary levels (e.g., hospitals, and clinical centers), particularly concerning medication reconciliation [54,55]. This recent implementation may explain why focus group participants did not mention the role of clinical pharmacists. In some health center, family doctors do collaborate with clinical pharmacists on medication reconciliation; however, the process still faces several challenges, including communication barriers between healthcare providers. The Slovenian healthcare system faces a number of challenges in providing continuous and well-coordinated care for people with complex post-hospital discharge care needs. The key challenge to enhancing continuity at the interfaces between care levels is a lack of standardization of processes and procedures. These challenges are further exacerbated by the lack of integrated information systems, which undermines the continuity of the healthcare process [29]. Forsyth et al. [21] stressed the importance of clear communication and a holistic approach to patient care. The relationship between patients and their caregivers should be nurtured, particularly between physicians and older patients [56]. Poor patient–provider relationships lead to insufficient patient counselling and leave the patient alone struggling with medication problems [57]. Better inter-professional collaboration and more open communication are important to overcome the problems of poor medication adherence in people with chronic conditions [35]. Krause et al. [58] emphasized the need for thorough communication between hospitals and general practitioners to ensure consistency in medication prescribing.

Several facilitative interventions were identified to improve medication adherence among the vulnerable elderly. Participants stressed the importance of regular medication review and regimen management. Personal physicians should oversee this process, supported by community nurses who can prepare medication lists and ensure compatibility with patients’ medical records. In Slovenia, community nurses may have daily contact with the patient and their family as part of their regular work, in coordination with the personal physician. They serve as the coordinators of all forms of home care and act as the link between the patient and their personal physician [59]. Simplified medication lists, color-coded for different times of the day, were suggested to help the vulnerable elderly follow their regimens. Dispensers with clear markings and having one person responsible for medication preparation can prevent errors [19,43]. Reducing the number of medications prescribed would appear optimal, although it is often not possible, as this has been seen to have an immediate positive impact [60]. Pharmacies providing simplified medication summaries could further aid in adherence [18].

Educating the vulnerable elderly and their informal caregivers about proper medication use is crucial. Physicians and community nurses play a key role in ensuring patients understand instructions regarding dosage, timing, and administration methods. patients’ education as a core of intervention is recommended to minimize patient myths about their treatment plans [3]. Health literacy programs should be developed, focusing on chronic disease management and medication adherence [21,52]. Educational materials should be clear, use simple language, and include large print in order to be accessible to the vulnerable elderly. Coskun et al. [52] emphasized the importance of clear communication and appropriate pedagogical methods.

A robust social support network is vital for medication adherence. Family and neighbors provide significant support, especially for those living alone. Maintaining good relationships and involving relatives in medication management can reduce the burden on vulnerable elderly patients. In rural areas, community-organized help is more common, while urban areas face more isolation. NGOs and patient associations offer companionship and practical support, sharing valuable information and experiences. Several studies support our findings about the multifaceted role that social support networks play in enhancing medication adherence. Holt et al. [50] underscored social support, such as family involvement and community resources, as a key component. Maffoni et al. [61] also highlighted the importance of social support in adherence. The relationship between healthcare providers and patients is critical, along with family and community support.

Ensuring continuity of care is vital for medication adherence and overall health outcomes in the elderly. Proper transfer of information between healthcare providers upon patient discharge, assisting patients in establishing contact with their personal physicians, and coordination among all involved in the patient’s care are crucial strategies. Dharvees et al. [18] highlight the importance of regular follow-ups and monitoring by healthcare providers to facilitate continuity of care. Krause et al. [58] emphasized that effective communication between hospital and primary care physicians can align prescribing practices and ensure consistency in patient care. Coordination and cooperation among healthcare providers and caregivers are crucial to address these factors effectively [20,56,61].

The findings of this study have significant implications for healthcare policy, practice, and patient education. By identifying and understanding the barriers and facilitators to medication adherence among the vulnerable elderly, we can develop more targeted interventions to improve health outcomes. Future research should focus on evaluating the long-term efficacy of these interventions to ensure sustained improvements in medication adherence and overall health in the vulnerable elderly. Effective interventions could include regular medication reviews, comprehensive educational programs, and the integration of digital health technologies. The social network of the vulnerable elderly needs to be strengthened and coordination and cooperation between all those involved in their care needs to be ensured. At a system level, there is a need to regulate the appropriate transfer of information between providers when a patient is discharged to his/her home environment, thus ensuring continuity of care.

This study has several limitations that should be considered. First, the use of focus groups, while valuable for in-depth qualitative insights, may limit the generalizability of the findings to broader populations. Second, the study relied on self-reported data, which can be subject to recall bias and social desirability bias. Third, the study was conducted in a specific cultural and healthcare context, which may limit the applicability of the findings to different regions or countries with varying healthcare systems and cultural practices regarding medication adherence and elderly care.

## 5. Conclusions

This study highlighted numerous barriers faced by the vulnerable elderly in medication adherence and facilitative interventions that could alleviate these problems. Some barriers can be directly influenced, others stem from the nature of illnesses and health conditions, and some require systemic solutions at the national or policy level. The vulnerable elderly need substantial support in enhancing health literacy and medication-related health literacy, as well as in medication preparation. It is also crucial to organize medication lists, assist in establishing contact with personal physicians, and ensure continuous healthcare provision upon hospital discharge. The importance of support networks for the vulnerable elderly living at home were emphasized. These findings are crucial for planning locally relevant interventions to improve medication adherence among the vulnerable elderly. Based on these findings, we have developed an intervention and evaluated it in a randomized control study.

## Figures and Tables

**Table 1:** The main categories and codes we have developed based on the results of the focus group interviews on barriers and facilitators to medication adherence among the vulnerable elderly.

Main Categories	Categories	Codes
Perceived Barriers	Medication-related barriers	Medication instructions, difficulties in procuring, switching, and replenishing medication supplies, purchasing non-prescription drugs and dietary supplements, polypharmacy/excessive medications, problems with improper medication handling and refusal/discontinuation of medication
	Patient-related barriers	Physical difficulties and conditions, decline in general fitness and cognitive abilities, health literacy and ICT literacy
	Barriers related to the healthcare system/healthcare personnel	Disorganized discharge from the hospital, (In)accessibility of a personal physician, disruption in continuity of care, communication
Facilitative interventions	Medication management	Medication review and regimen management, medication dispensers/medication lists
	Health education	Health education work with patients, relatives/significant others/close ones, improve health literacy and medication-related health literacy
	Supportive social networks	Involvement of relatives and significant others (close ones), healthcare providers, patient associations and NGOs
	Ensuring continuity of care	Arrange proper transfer of information between providers upon patient discharge to home/ensure continuity of care, assist patients in establishing contact with their personal physician, coordination and cooperation of all involved in the care of the vulnerable elderly

## Data Availability

Data are available upon request from the corresponding author.

## Supplementary Materials

The following supporting information can be downloaded at: https://www.mdpi.com/article/10.3390/healthcare12171723/s1, File S1: Interview guide.
