# Supplementary material for: Barriers and Facilitators to Medication Adherence among the Vulnerable Elderly: A Focus Group Study
Source: Healthcare (Basel). 2024 Aug 29;12(17):1723. doi: 10.3390/healthcare12171723 (PMC11395048; doi:10.3390/healthcare12171723)
Supplement: Supplementary file 1 [file healthcare-12-01723-s001.zip › healthcare-3136371-supplementary.pdf]

Supplementary Materials File S1

Interview Guide

How do you see your role in addressing the individual and family within the framework of home care services?

What does your profession mean to you? Can you also provide an example?

What vulnerabilities do you observe in your work with older adults?

What differences do you notice between rural and urban environments?

How often do you encounter problems with older individuals failing to take their prescribed medications as directed by a doctor in your work/care for family members?

What are the most common issues related to taking prescribed medications as directed by a doctor that older people you visit as part of your work/family care face?

In your opinion, how well do older people whom you visit as part of your work/care for in the family, know their prescribed medications (effects, regimen)?

What factors make it difficult for older people whom you visit as part of your work/care for in the family to take prescribed medications as per doctor's instructions?

What factors facilitate the taking of prescribed medications as per doctor's instructions by older people whom you visit as part of your work/care for in the family?

How successful, in your opinion, are the older people whom you visit as part of your work/care for in the family at self-monitoring their condition?

Which older people have the most trouble taking prescribed medications as per doctor's instructions?

How could you help them overcome these difficulties?

Who could help them overcome these difficulties?

Is there anything else you would like to say about the barriers to medication adherence encountered by older people, which we have not asked you about?
